# Ionic Liquids Separating Rubber Latex from Guayule

**DOI:** 10.3390/ma14154255

**Published:** 2021-07-30

**Authors:** Joan G. Lynam, Holden T. Zugger, Elizabeth T. Amedee

**Affiliations:** Department of Chemical Engineering, Louisiana Tech University, 818 Nelson Ave, Ruston, LA 71272, USA; holdenzugger@yahoo.com (H.T.Z.); elizamedee@gmail.com (E.T.A.)

**Keywords:** *Hevea*, *Parthenium*, FTIR, solvent, centrifuge

## Abstract

Danger to rubber trees (*Hevea brasiliensis*) from South American leaf blight fungus imperils the world’s source of natural latex for essential rubber products. Avoiding latex allergies also requires a non-Hevea latex source. The present methods for removing latex entrapped in the individual cells of guayule plants require environmentally hazardous chemicals. This study proposes a new method for latex extraction from guayule using various ionic liquids (ILs) to dissolve cell walls and release latex, as substantiated by Fourier transform infrared spectroscopy (FTIR) data.

## 1. Introduction

Rubber is an essential part of modern economies. Rubber from natural sources is superior in tensile strength and other properties to synthetic rubber and also is not derived from fossil fuels [1,2]. Rubber trees (*Hevea brasiliensis)* originated in the Amazon basin in South America but have been planted in many tropical regions beginning in 1876. However, South American leaf blight (*Microcyclus ulei)*, caused by a fungus, has devastated rubber trees in South America, so that there is presently no commercial-scale natural rubber production there. This fungus has the potential to be distributed in other areas where rubber trees are grown, eventually causing a worldwide shortage of natural rubber latex. Other leaf blights threaten Southeast Asia rubber trees, as well [3]. Non-rubber tree latex-containing plants, such as guayule (*Parthenium argentatum*), are a possible substitute for latex from *Hevea brasiliensis* if such a crisis should occur. Guayule latex, in particular, also has advantages over rubber tree latex in that it is non-allergenic [4,5]. Other plants, such as *Taraxacum kok-saghyz* (Russian dandelion), can also be used to produce rubber, but guayule has an advantage in that it thrives in an arid climate with marginal soil [6,7].

Latex rubber from non-rubber tree plants, such as guayule, is contained within the plant cells, requiring the breaking of parenchyma cell walls to release the latex suspended in the aqueous cytoplasm [8]. Typically, grinding/milling at a pH of 10 is employed to break cell walls and release latex [9]. Alternatively, volatile organic solvents, both polar or non-polar, may be used to release latex [10]. The process waste streams, which are either high pH or comprised of volatile organic compounds, may be devastating when released into the environment [9]. A more environmentally friendly process is vitally needed. 

Ionic liquids (ILs) are defined as water-free organic salts with melting points at temperatures below their decomposition temperature [11]. They have been reported to break down cell walls in plants [12,13]. Their large cations cause the poor organization of cations with anions, thus lowering these solvents’ melting points. As they are salts, ILs have extremely low vapor pressures, which facilitates recycling and makes them “green” when they are used in biomass pretreatment. Some ILs have been reported to be of low hazard to the environment, such as 1-ethyl-3-methylimidazolium acetate (Emim Ac), a low toxicity IL (LD50 > 2000 mg kg^−1^) [14]. If halogens and sulfur are avoided when synthesizing ILs, then their decomposition products are reported to be relatively harmless [15]. Nevertheless, full life cycle analyses need to be conducted even on these ILs [16]. Further advanced ionic liquids, such as deep eutectic solvents, may also be even more ecofriendly for biomass deconstruction [17]. The present work investigates the effect of various ionic liquids on the removal of latex from guayule stems. Our hypothesis is that a process using an environmentally friendly IL can be found to release latex from guayule, as indicated by FTIR analysis of the products obtained. 

## 2. Materials and Methods

### 2.1. Materials

Guayule stems were collected from two-year-old plants of improved germplasm (CAL-7), generously provided by Dr. Hussein Abdel-Haleem from the US Arid-Land Agricultural Research Center at Maricopa Agricultural Center, Maricopa, AZ, USA. CAL-7 was developed by the University of California and released for its high rubber content [14,15]. It has a rubber content of approximately 10% [16]. The stems were stored in the refrigerator prior to use. Wire cutters were used to chop the stems; a blender (SharkNinja Operating LLC, Chino, CA, USA) was used to grind the pieces. Lastly, the ground guayule was sieved between 14 and 20 count mesh sieves to obtain particles of approximately 1 mm in diameter. The ILs 1-ethyl-3-methylimidazolium acetate >95% (Emim Ac), 1-ethyl-3-methylimidazolium lactate >98% (Emim Lact), 1,3-dimethylimidazolium dimethylphosphate >98% (Dmim Dmp), and 1-ethyl-3-methylimidazolium diethylphosphate >98% (Emim Dep) were purchased from IoLiTec, Inc. [18] and used as received. Recycling of ionic liquids was reported as a means of re-using these materials [19,20]. Centrifuged Natural Latex Liquid Rubber for comparison was purchased from Holden’s Latex Corporation (New York, NY, USA).

### 2.2. Methods

Guayule (2 g) was mixed with 20 g of the IL for a 1:10 mass ratio. The capped glassware with the mixture was immersed in an oil bath at temperatures of 110, 125, or 140 °C with magnetic stirring. Samples were taken at 30, 60, 90, and 120 min. After sampling, approximately 7 g of water was added to each sample in a tube. The tubes were vortexed and then centrifuged for 10 min at about 1000 RPM. The layers obtained were separated by pipetting prior to Fourier Transform Infrared (FTIR) spectroscopy. Latex clumps were separated by vacuum filtration. Each sample was analyzed using a Nicolet IR 100 FTIR-ATR (Thermo Scientific, Waltham, MA, USA) with a diamond crystal using 32 scans per sample and a resolution of 2.0 for frequencies of 700 to 4000 cm^−1^. Spectra were obtained under ambient lab conditions (22 °C, 1 atm).

## 3. Results

**Figure 1:**
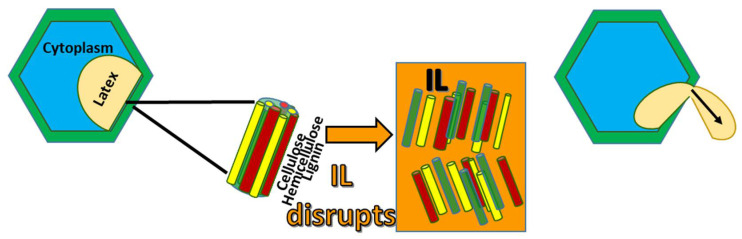
Schematic of Ionic Liquid Action.

**Figure 2:**
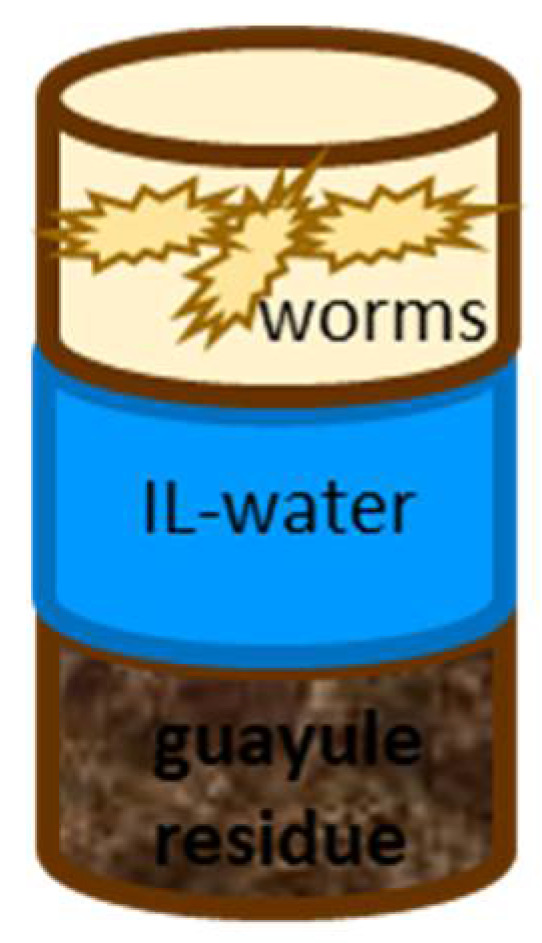
Schematic of Layers after Ionic Liquid Pretreatment.

The effect of ionic liquid expected on cell walls is shown in Figure 1. After pretreatment with the various ionic liquids, and the addition of water, either two or three layers formed and became even more distinct with centrifugation. All two or three layers were separately characterized by FTIR. The top layer was observed to contain clumps, historically called worms. The top layer would be expected to be rubber latex, since its density was measured as 0.95 g/cm^3^, while the density of water was 1.0 g/cm^3^ and the density of Emim Ac was measured at 1.1 g/cm^3^ [14]. Figure 2 shows a schematic of the layers observed. Samples from the top layer after centrifugation showed the relevant vibrations but varied in intensity with reaction conditions. Lower layers after centrifugation showed only peaks for the IL or the original biomass. 

The most relevant and characteristic IR vibrations for guayule latex were reported to be near 740 cm^−1^, 830 cm^−1^, 1370 cm^−1^, 1440 cm^−1^, and 1655 cm^−1^ [21]. For natural rubber, the 742 cm^−1^ vibration was attributed to a rocking –CH2– bond that would exist if the latex bonds to an adjacent isoprene [22]. A vibration corresponding to the backbone C–H out-of-plane mode (C=C–H) at 836 cm^−1^ shifted to 843 cm^−1^ when under strain, as would exist with FTIR-ATR measurements [23]. 

A 1037 cm^−1^ vibration was assigned to a rocking –CH_3_ group, a 1245 cm^−1^ vibration to a twisting –CH_2_– bond, a 1375 cm^−1^ vibration to a –CH_3_ deformation, and a 1447 cm^−1^ vibration to a –CH_2_– deformation or a rocking –CH_3_ group [22]. A 1654 cm^−1^ vibration was assigned to C=C stretching [24]. The relevant vibrations and their assignments can be seen in Table 1. Vibrations for latex that overlap with the IL, such as 1090 cm^−1^ (a twisting –CH_2_–), were not analyzed or discussed since conclusions cannot be drawn from these data.

**Figure 3:**
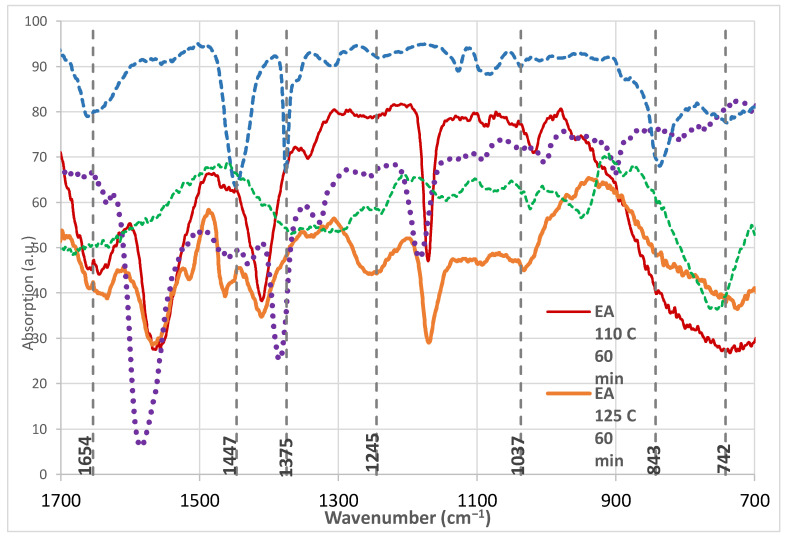
FTIR spectra in the fingerprint region of commercial latex, guayule pretreated with Emim Ac at 110 °C for 60 min (EA 110 C 60 min), guayule pretreated with Emim Ac at 125 °C for 60 min (EA 125 C 60 min), Emim Ac, and untreated guayule.

The “worms” in the top layer after centrifugation showed the relevant vibrations, although they varied in intensity with reaction conditions. Lower layers after centrifugation showed only peaks for the IL or the original biomass. As seen in Figure 3 and Table 1 for Emim Ac pretreatment, all latex vibrations exist for a 60 min pretreatment at 110 °C and at 125 °C, but shorter or longer pretreatment times show weaker characteristic vibrations. In these Emim Ac pretreated spectra in Figure 3, a sharp vibration was seen at 1170 cm^−1^, which reflects contamination with Emim Ac. At the highest temperature, 140 °C, the most characteristic vibrations are seen at the shortest time of 30 min. These findings suggest that the severity of IL pretreatment can affect the latex produced. Too long a time at high temperature may cause degradation of the latex, while too short a time does not degrade the cell wall enough to allow the latex to escape.

When guayule was pretreated with 1-ethyl-3-methylimidazolium lactate (Emim Lact), all the latex vibrations that did not overlap with the Emim Lact spectra were observed at the conditions 125 °C for 30 min, but longer times of 60 min, 90 min, and 120 min showed fewer common vibrations. Again, higher pretreatment severity likely decomposed latex.

For 1-ethyl-3-methylimidazolium diethylphosphate (Emim Dep), pretreatment at 125 °C 30 min, 60 min, and 90 min showed latex vibrations, but not 120 min. For 1,3-dimethylimidazolium dimethylphosphate (Dmim Dmp) pretreatment at 125 °C, 60 min of pretreatment showed more latex vibrations compared to 30 min, 90 min, and 120 min, showing 60 min to be the optimal time for pretreatment with this IL.

## 4. Discussion

Analyses of FTIR spectra from IL pretreatment of guayule suggest that cell walls are broken, allowing the rubber latex inside to escape. The addition of water leads to the formation of layers, with the lowest density top layer showing characteristic rubber latex vibrations in the FTIR spectra. The bottom layers shows the spectral characteristics of IL or biomass residue. Cellulose would be expected to remain in the residue in the lower layers, since the ILs used tend to sparingly dissolve cellulose [25]. A more environmentally friendly method for the separation of rubber latex from guayule may then be possible, permitting it to become a backup source of natural rubber latex, should *Hevea brasiliensis* succumb to disease in Asia. Future studies of cell wall breakage of guayule and other rubber-containing plants using advanced ionic liquids could allow for even more environmentally friendly solutions to processing plants for rubber products. In addition, future work is needed to compare this method to conventional methods and to find if latex rubber is contaminated with IL, although Emim Ac has been reported to be harmless to aquatic life [26]. Future work of a careful accounting of IL solvent and the ability to recycle it will be needed to justify the economics of this method.

## Figures and Tables

**Table 1:** Vibrations Common to Guayule Latex and Guayule Pretreated with Emim Ac.

Latex Wave-Number (cm^−1^)	1654	1447	1375	1245	1037	843	742
Bond Vibration	C=C stretching	–CH_2_– deformation/rocking –CH_3_	–CH_3_ deformation	twisting –CH_2_–	rocking –CH_3_ group	backbone C–H out-of-plane mode	rocking –CH_2_–
110 °C/30 min	✓		✓	✓	✓		✓
110 °C/60 min	✓	✓	✓	✓	✓	✓	✓
110 °C/90 min	✓	✓	✓	✓		✓	
110 °C/120 min	✓	✓	✓	✓	✓	✓	✓
125 °C/30 min	✓			✓	✓		
125 °C/60 min	✓	✓	✓	✓	✓	✓	✓
125 °C/90 min	✓	✓	✓		✓	✓	✓
125 °C/120 min		✓	✓		✓		✓
140 °C/30 min	✓	✓		✓	✓		✓
140 °C/60 min	✓		✓	✓	✓		✓
140 °C/90 min	✓	✓		✓	✓		✓
140 °C/120 min	✓			✓			✓

## Data Availability

The data presented in this study are available within this article.
